# Associations between Thyroid Hormones and Cognitive Impairment in Patients with Parkinson's Disease

**DOI:** 10.1523/ENEURO.0239-24.2024

**Published:** 2024-10-01

**Authors:** Yingying Peng, Lan Zhu, Qingling Bai, Limin Wang, Qian Li

**Affiliations:** ^1^Departments of Neurology, Cangzhou Central Hospital, Cangzhou 061000, China; ^2^Neurosurgery, Cangzhou Central Hospital, Cangzhou 061000, China

**Keywords:** cognitive impairments, Parkinson's disease, thyroid hormone

## Abstract

This study aims to explore the correlation of serum thyroid hormone levels to cognitive impairments in Parkinson's disease (PD) patients. In this retrospective study, 106 Chinese patients without cognitive impairments and 94 patients with cognitive impairments, including 55 with mild cognitive impairment (PD-MCI) and 39 with PD dementia (PDD), were analyzed. Clinical data regarding the PD assessments, including disease duration, Unified Parkinson's Disease Rating Scale (UPDRS) Part 3 scores, and Hoehn and Yahr (H–Y) staging, were analyzed. Cognitive functions were evaluated using the Montreal Cognitive Assessment score. Serum levels of thyroid-stimulating hormone (TSH), free thyroxine (FT4), and free triiodothyronine (FT3), were measured using ELISA. Significantly altered H–Y staging, disease duration, and UPDRS Part 3 scores were observed in PD patients with cognitive impairment compared with those without. Serum levels of FT3 were significantly decreased, while FT4 and TSH levels were significantly elevated in PD patients with cognitive impairment compared with those without. Combined detection of TSH, FT3, and FT4 showed value in distinguishing PD patients with and without cognitive impairment. Furthermore, a comparison of serum levels between PD-MCI and PDD patients revealed significant association between thyroid hormone levels and the degree of cognitive impairment in PD patients. Our findings suggest a relationship between changes in serum thyroid hormone levels and cognitive impairments in PD patients. Thyroid hormone levels, particularly FT3, may serve as potential markers for cognitive dysfunction in PD.

## Significance Statement

The correlation of serum thyroid hormone levels to cognitive impairments in Parkinson's disease (PD) patients remains unclear. The current results demonstrate a relationship between changes in serum thyroid hormone levels and cognitive impairments in PD patients. Our findings suggest that thyroid hormone levels, particularly free triiodothyronine, may serve as potential markers for cognitive dysfunction in PD.

## Introduction

Parkinson's disease (PD) is a neurodegenerative disorder distinguished by a constellation of motor symptoms including rigidity, postural instability, bradykinesia, and resting tremor, with the hallmark pathological features being progressive degeneration of the nigrostriatal dopaminergic system and the formation of Lewy bodies in neurons (Balestrino and Schapira, 2020). While the diagnosis and treatment of PD primarily emphasize motor symptoms, growing evidence suggests that PD is a multisystem syndrome involving nonmotor manifestations beyond dopamine deficiency in the nigrostriatal system (Tolosa et al., 2021). Nonmotor symptoms exhibited in PD encompass autonomic dysfunction, neuropsychiatric symptoms, sensory symptoms, gastrointestinal symptoms, and sleep disorders (Blesa et al., 2022). Among these, cognitive impairments bear considerable significance, not only contributing to functional decline but also entailing a tangible toll on individuals, families, and society, potentially culminating in mortality (Aarsland et al., 2021). However, the underlying mechanisms contributing to the wide range of motor and nonmotor symptoms remain unclear. Currently, several potential molecular and cellular mechanisms, including infection (Smeyne et al., 2021), environmental and genetic susceptibility (Tran et al., 2020), mitochondrial dysfunction (Malpartida et al., 2021), autophagy impairment (Hou et al., 2020), and neuroinflammation, have been implicated in neurodegeneration associated with PD (LeWitt and Chaudhuri, 2020).

Thyroid hormones are crucial neuromodulators in the central nervous system (CNS; Mohammadi et al., 2021; Pagnin et al., 2021). The impact of thyroid hormones on the CNS extends from neuronal development in fetal stages to adulthood. Thyroid hormones hold paramount importance in the normal development, metabolism, and growth of various organ systems, including the CNS, in mammals. Clinical thyroid disorders have been identified as potential causes of neuropsychiatric symptoms and secondary or reversible dementia (George et al., 2019; Jurado-Flores et al., 2022). Recent studies have indicated that thyroid dysfunction [i.e., normal-range variation in thyroid-stimulating hormone (TSH) levels with free thyroxine (FT4) and free triiodothyronine (FT3)] and even variations within the normal range of thyroid function can affect cognitive function (Emerenziani et al., 2019; West et al., 2020). Specifically, changes within the normal range of serum TSH levels are found to be correlated with alterations in cognitive function (Santhanam et al., 2022).

The correlation between PD and thyroid dysfunction in terms of clinical manifestations and pathogenesis has been widely reported. However, our focus is on cognitive impairment related to PD. For example, it has been found that thyroid hormone levels and structural parameters of thyroid homeostasis are correlated with motor subtype and disease severity in euthyroid patients with PD, but this study did not address PD-related cognitive impairment (Tan et al., 2021). Similarly, Umehara et al. (2015) discovered that thyroid hormone levels, especially FT3 levels, are closely related to motor symptoms in patients with de novo PD, also without focusing on cognitive impairment associated with PD. Our work, on the other hand, is centered on the relationship between changes in serum thyroid hormones and cognitive impairment in PD patients.

Herein, our study aims to investigate the relationship between changes in serum thyroid hormone levels and cognitive impairments in PD patients. In this retrospective study, we analyze FT3, FT4, and TSH levels in PD patients with mild cognitive impairment (PD-MCI) or without or PD dementia (PDD), evaluating the correlation of these hormone levels to score of cognitive assessments.

## Materials and Methods

### Ethics statement

The study was approved by the ethics commitment of Cangzhou Central Hospital, the approval number is 2010-ZLL-09. The study was performed in strict accordance with the Declaration of Helsinki, Ethical Principles for Medical Research Involving Human Subjects.

### Informed consent

The written informed consent was obtained from all the participants of this study.

### Participants

This retrospective study included patients diagnosed with PD (103 males and 97 females) in accordance with the diagnostic criteria for PD in China (2016 version) during 2020–2022 in our hospital. The study was approved by the ethics commitment of Cangzhou Central Hospital (2010-ZLL-09). We acquired informed consent from all patients prior to analyze their data for the study.

Patients with secondary parkinsonism, resulting from conditions such as head trauma, infectious encephalitis, cerebrovascular accidents, accidental poisoning, etc., were excluded from the study. Patients with Parkinson's plus syndromes, including progressive supranuclear palsy and multiple system atrophy, were also excluded. Furthermore, patients with severe cardiac, hepatic, renal, or gastrointestinal dysfunctions were excluded, as well as those who demonstrated uncooperative behavior during neurological examinations and blood tests. Patients with autoimmune diseases and a history of thyroid diseases, individuals receiving thyroid hormone replacement therapy, or those taking anti-thyroid medications were also excluded. Additionally, patients with cognitive impairments caused by Alzheimer's disease, psychiatric disorders, or vascular dementia, which could potentially affect cognitive function, were excluded from the study.

### Enzyme-linked immunosorbent assay

Serum S100β, a member of the S100 protein family, mainly present in glial cells and Schwann cells, was detected using an ELISA kit (item number, EH0543; Wuhan Fine Biotech).

### Unified Parkinson's Disease Rating Scale (UPDRS)

The motor impairments in PD patients were assessed using Part 3 of UPDRS (Bernard et al., 2020). Part 3 primarily consists of Items 18–31, which are designed to evaluate motor impairments in PD patients. These assessments were conducted by physicians who had received training in the administration of the UPDRS.

### Disease severity classification using Hoehn and Yahr (H–Y) staging

The disease severity of PD patients was assessed and classified using the widely recognized H–Y staging system (Simon-Gozalbo et al., 2020). This staging system provides a standardized framework for evaluating the progression and degree of PD severity. The H–Y staging system consists of five distinct stages that represent different levels of functional impairment and disability experienced by patients.

### The Montreal Cognitive Assessment (MoCA)

The MoCA (Jia et al., 2021) was utilized to evaluate cognitive domains, including attention and concentration, memory, executive functions, visuospatial and constructional skills, language, abstract thinking, calculation, and orientation. The MoCA provides a total score out of 30, with an extra point added to the total score for those with education of <12 years (if the score is <30). Cognitive impairments were defined as a MoCA score below 26. In this study, PD-MCI patients were identified as those having a MoCA score below 26 but not meeting the criteria for PDD. Diagnosing PDD was based on the criteria outlined in the Chinese diagnostic and treatment guidelines for PDD and a MoCA score below 21.

### Evaluation of thyroid function

The serum concentrations of T4 and T3 are predominantly bound to thyroid-binding globulin, transthyretin, and albumin. However, only a minute fraction of T4 (0.02%) and T3 (0.3%) exist in their free, unbound state. To obtain a more precise assessment of T4 and T3 levels, it is imperative to measure the concentrations of FT4 and FT3, which allow for an evaluation independent of binding proteins. Moreover, FT4 and FT3 exhibit distinctive functions in various target tissues. Consequently, the measurement of FT4 and FT3 has superseded the assessment of total hormone levels encompassing both free and bound forms. In this investigation, fasting blood was collected, and the serum concentrations of FT3, FT4, and TSH were quantified using Roche reagents on the Cobas e601 electrochemiluminescence immunoassay analyzer.

### Statistical test

The required sample size was calculated with *n* = (*Zα*/2 × *σ*/*E*)2 (*n* is the sample size, *Zα*/2 is the degree of confidence, *σ* is the standard deviation, and *E* is the margin of error). Estimates of effect size and standard deviation were based on the existing literatures. We assumed *α* = 0.05 and *β* = 0.2. GraphPad Prism was used for analysis. Categorical data were presented as *n* (percentage) or median (interquartile range) and subjected to analysis using Fisher's exact test or the Mann–Whitney test. Numerical data were visually represented through the utilization of box plots. To determine the statistical significance, the following designations were employed, *p < 0.05 and ***p < 0.001, ascertained using the unpaired *t* test with Welch's correction. Pearson's correlation analysis was employed to evaluate the relationships between two parameters, yielding the correlation coefficient (*r*) and the corresponding *p* value.

## Results

### Study design and baseline clinical characteristics

In this study, a cohort of 200 eligible individuals diagnosed with PD was carefully selected as the study participants. Among them, 106 patients exhibited no signs of cognitive impairments, while the remaining 94 patients displayed cognitive impairments, encompassing 55 PD-MCI patients and 39 individuals diagnosed with PDD. Baseline analysis of the clinical data indicated significant disparities between PD patients without cognitive impairments (PD) and those with cognitive impairments (PDCI). Notably, notable differences were observed in disease duration (*p* = 0.010), H–Y staging (*p* = 0.008), UPDRS Part 3 scores (*p* = 0.004), and MoCA scores (*p* < 0.001) between the two cohorts. Conversely, factors such as age, gender, smoking (Behl et al., 2024), presence of comorbidities like diabetes mellitus and hypertension, as well as educational background exhibited no significant distinctions between the two groups (Table 1). Notably, in the multivariate analysis presented in Table 2, gender was also not an independent risk factor in our sample. Therefore, we did not consider the confounding effect of gender.

**Table 1.** Demographic and clinical characteristics of PD and PDCI patients

Features	PD (*n* = 106)	PDCI (*n* = 94)	*p* value
Age (years)	65 (59, 70)	67 (61, 71)	0.155
Course of PD (months)	28.5 (16.75, 40)	35 (23, 46.25)	0.010
Gender			
Male	51 (48.1%)	52 (55.3%)	0.198
Female	55 (51.9%)	42 (44.7%)	
Smoke			
Yes	34 (32.1%)	38 (40.4%)	0.239
No	72 (67.9%)	56 (59.6%)	
Diabetes mellitus			
Yes	18 (17.0%)	24 (25.5%)	0.165
No	88 (83.0%)	70 (74.5%)	
Hypertension			
Yes	35 (33.0%)	41 (43.6%)	0.145
No	71 (67.0%)	53 (56.4%)	
Education			
Junior high school and below	47 (44.3%)	50 (53.2%)	0.257
High school and above	59 (55.7%)	44 (46.8%)	
H–Y stage	2 (1.25, 2.75)	3 (1.75, 4)	0.008
UPDRS Part 3 score	30 (23.75, 37)	34.5 (26.75, 42.25)	0.004
MoCA	28 (27, 29)	20 (17, 22.25)	<0.001

The data are presented as *n* (percentage) or median (interquartile range). The comparisons of data were done by the Mann–Whitney test or Fisher's exact test. MCI, mild cognitive impairments; PDD, PD dementia; UPDRS, Unified PD Rating Scale.

**Table 2.** Multivariate logistic analysis of clinicopathological factors for cognitive impairments in PD patients

	OR	95% CI	*p* value
Course of PD	1.84	1.22–3.05	0.002
Serum FT3	1.26	1.04–1.77	0.021
Serum FT4	1.31	1.12–2.03	0.018
Serum TSH	1.35	1.09–2.56	0.009

OR, odds ratio.

### Serum levels of FT3, FT4, and TSH in PD patients with and without cognitive impairment

**Figure 1.**
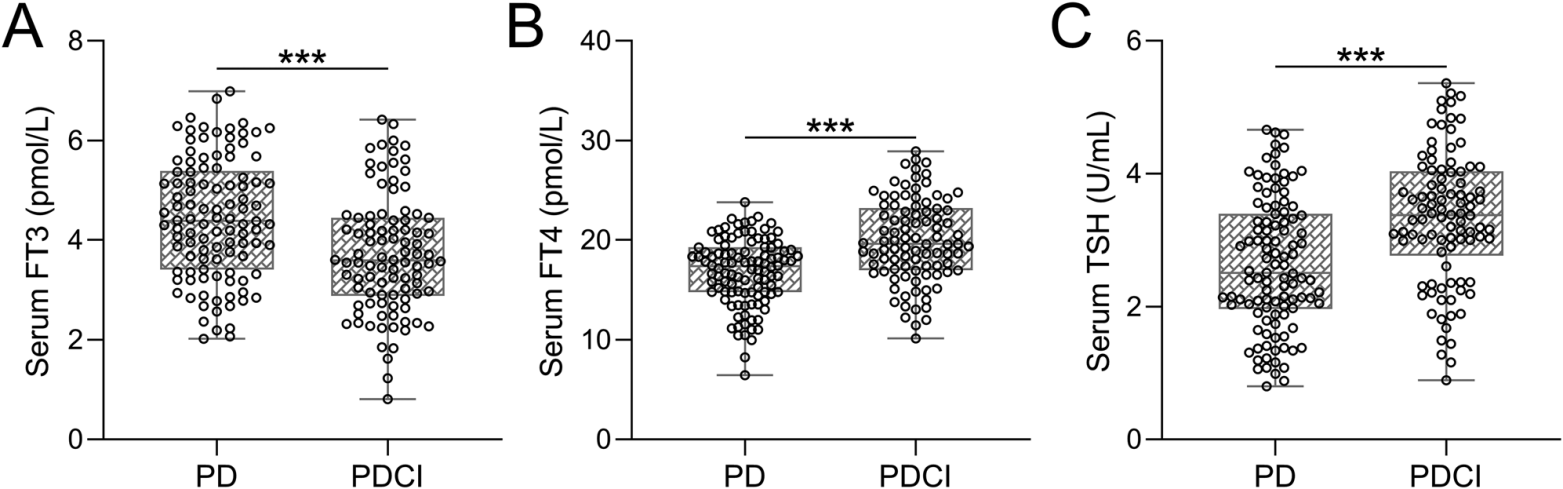
Comparative analysis of serum levels of FT3 (***A***), FT4 (***B***), and TSH (***C***) in PD patients without cognitive impairments (PD, *n* = 106) and PDCI patients (*n* = 94).

Given the modulatory effects of thyroid hormones on cognitive function, we undertook a comparative analysis of serum levels of key thyroid hormones, including FT4, FT3, and TSH, between PD patients lacking cognitive impairment (PD) and those burdened with cognitive impairment (PDCI) (Fig. 1). Remarkably, the PDCI group displayed notably lower FT3 levels (Fig. 1*A*) while exhibiting significantly higher FT4 (Fig. 1*B*) and TSH (Fig. 1*C*) levels in comparison with the PD group (*p* < 0.001).

### Diagnostic value of FT3, FT4, and TSH in discriminating PD patients with and without cognitive impairment

**Figure 2.**
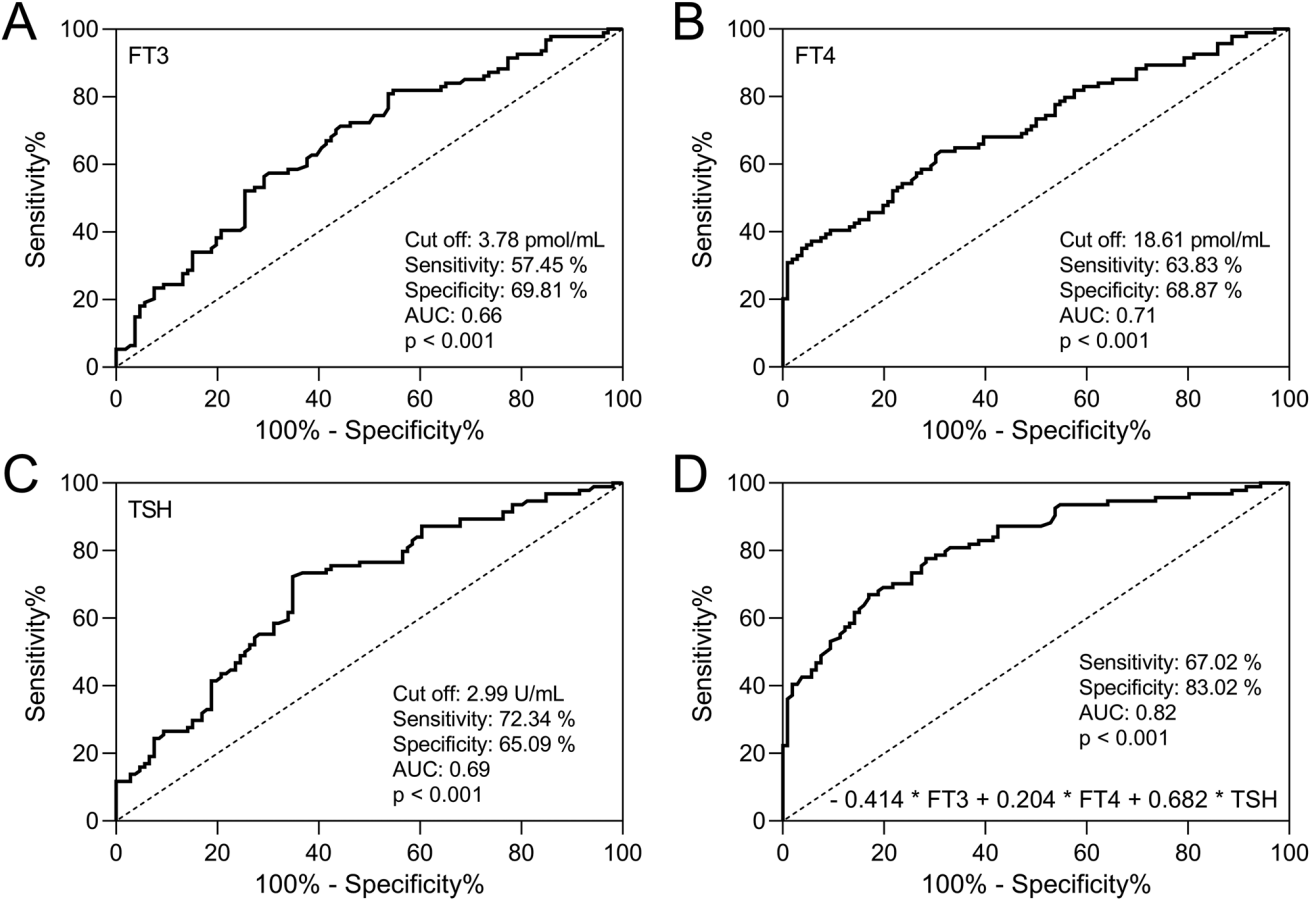
Receiver operating characteristic (ROC) curves illustrating the performance of serum FT3 (***A***), FT4 (***B***), TSH (***C***), and their combined test (***D***) in differentiating PDCI patients.

The striking alterations in thyroid hormone levels among PD patients afflicted with cognitive impairment compelled us to explore the diagnostic potential of these hormones in assessing the risk of cognitive decline. Figure 2 effectively illustrates the discriminative value of individually assessing FT3 (Fig. 2*A*), FT4 (Fig. 2*B*), and TSH (Fig. 2*C*) through receiver operating curve analysis, as well as the combined detection factor (Fig. 2*D*). The combined detection factor, calculated using the equation −0.414 * FT3 + 0.204 * FT4 + 0.682 * TSH, exhibited the highest discriminatory capability (sensitivity, 67.02%; specificity, 83.02%; AUC, 0.82; *p* < 0.001). Interestingly, all the hormones displayed moderate sensitivity and specificity (AUC of 0.66–0.71).

### Comparison of serum levels of FT3, FT4, and TSH in PD-MCI and PDD patients

**Figure 3.**
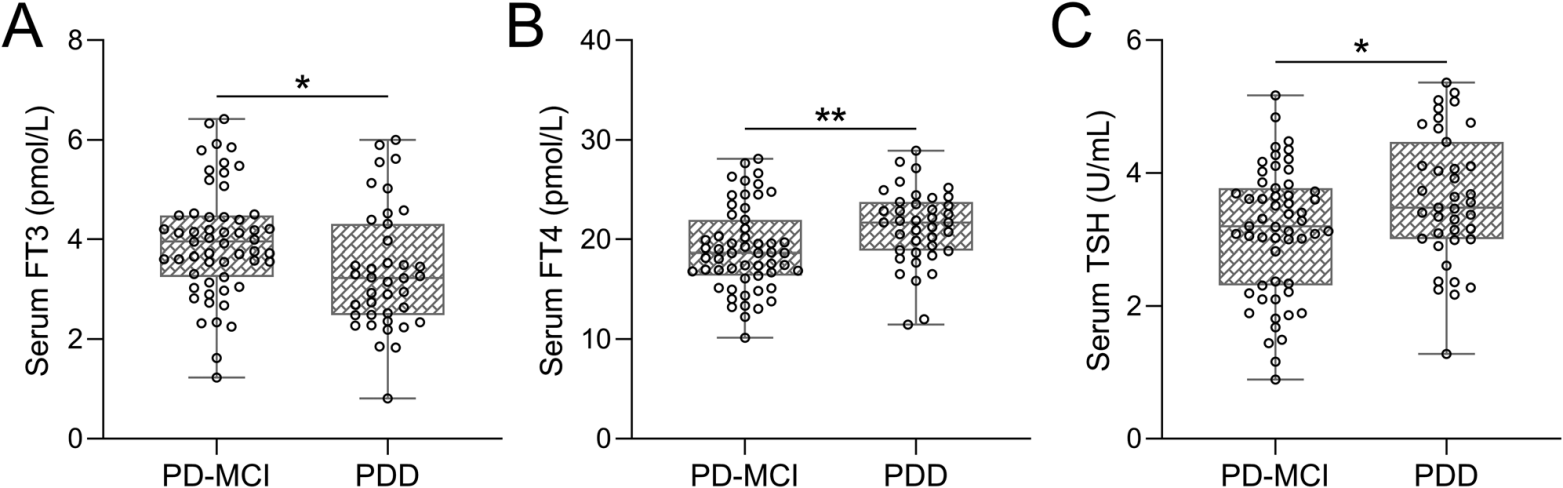
Comparative evaluation of serum FT3 (***A***), FT4 (***B***), and TSH (***C***) levels between PD-MCI patients (*n* = 55) and PDD patients (*n* = 39).

Further exploration of the diagnostic potential of thyroid hormones was undertaken to distinguish between PD-MCI and PDD. Consistent with the understanding that PDD is characterized by greater severity compared with PD-MCI, notable disparities were detected, with significantly lower FT3 levels (Fig. 3*A*) and elevated FT4 (Fig. 3*B*) and TSH (Fig. 3*C*) levels observed in the PDD group in comparison with the PD-MCI group. These findings provide additional support for the association between thyroid hormone levels and the severity of cognitive impairment in PD patients.

### Correlation between S100β, MoCA, and MMSE scores and serum levels of FT3, FT4, and TSH in PDCI patients

**Figure 4.**
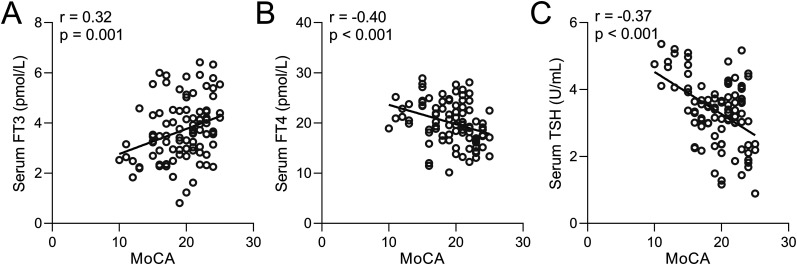
Spearman correlation analysis examining the relationship between MoCA scores and serum levels of FT3 (***A***), FT4 (***B***), and TSH (***C***) in PDCI patients (*n* = 94).

**Figure 5.**
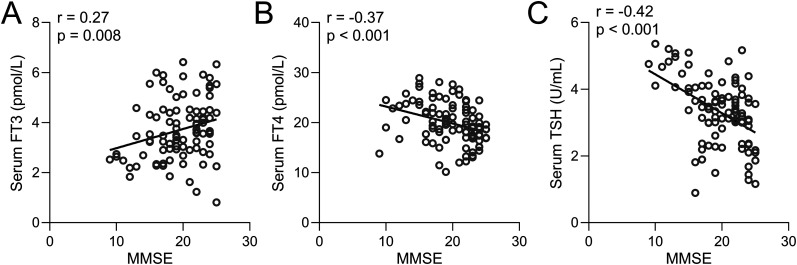
Spearman correlation analysis examining the relationship between MMSE scores and serum levels of FT3 (***A***), FT4 (***B***), and TSH (***C***) in PDCI patients (*n* = 94).

**Figure 6.**
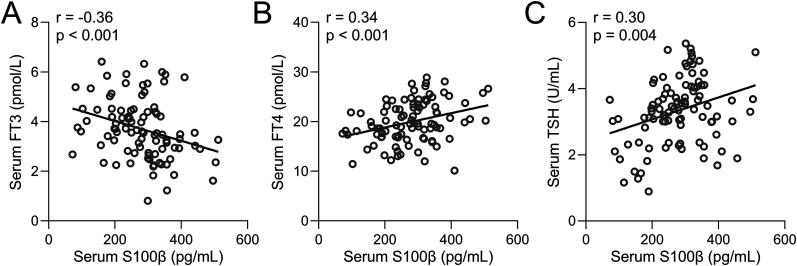
Spearman correlation analysis examining the relationship between serum S100β and serum levels of FT3 (***A***), FT4 (***B***), and TSH (***C***) in PDCI patients (*n* = 94).

In order to elucidate the relationship between thyroid hormone levels and cognitive performance, a correlation analysis was performed between MoCA scores and serum levels of FT3 (Fig. 4*A*), FT4 (Fig. 4*B*), and TSH (Fig. 4*C*) specifically among PDCI patients. The findings revealed a positive correlation between MoCA scores and FT3 levels, as well as a negative correlation between MoCA scores and FT4 and TSH levels. These results further substantiate the connection between thyroid hormone levels and the severity of cognitive impairment in PD patients afflicted with cognitive decline. Similarly, minimental state examination (MMSE) scores also showed significant correlations to FT3 (Fig. 5*A*), FT4 (Fig. 5*B*), and TSH levels (Fig. 5*C*) in PDCI patients. We also found that the conventional PD marker, S100β, which increases with damaged BBB, was also negatively correlated to FT3 (Fig. 6*A*) but positively correlated to FT4 (Fig. 6*B*) and TSH (Fig. 6*C*).

## Discussion

Cognitive impairments in PD significantly contribute to the loss of normal functioning and overall patient prognosis (Fang et al., 2020). Therefore, identifying potential biomarkers associated with cognitive dysfunction is of great clinical importance. Thyroid dysfunction has been shown to affect cognitive function in both PD and non-PD populations (Schneider et al., 2023), which prompted us to investigate the relationship between serum thyroid hormone levels and cognitive impairments in PD patients. In this study, we observed significant differences in serum levels of FT4, FT3, and TSH between PD patients with and without cognitive impairment. Our findings provided definite proof that changes in thyroid hormone levels have clinical implications for cognitive outcomes, potentiating the use of alterations in thyroid hormone levels as new biomarkers in the development and progression of cognitive impairments in PD. Furthermore, the correlation of thyroid hormones to the severity of cognitive impairment in PD, i.e., capability of differentiating PD, PD-MCI, and PDD in Figure 3, suggests the use of these hormone levels as quantitative indicators to tailor management for PD patients.

Hence, our study fosters the development of a clinical tool for assessing cognitive impairment among PD patients which bears unique challenges compared with that among other patient population (Sun and Armstrong, 2021). First, cognitive impairment in PD can overlap with other nonmotor symptoms, such as depression, apathy, and fatigue (Jenner et al., 2020). These symptoms can contribute to cognitive difficulties and may make it difficult to distinguish between cognitive impairment due to PD and cognitive impairment arising from other factors. In addition, PD is a progressive neurodegenerative disorder, and cognitive impairment can worsen over time. Furthermore, many cognitive assessment tools commonly used in clinical practice were initially developed for other populations, such as Alzheimer's disease or general cognitive impairment. These tools may not be sensitive enough to capture the unique cognitive profile and subtle changes associated with PD-related cognitive impairments. Finally, the serum biomarkers portend the use of a clinical tool desirable to long-term monitoring of cognitive function of PD patients without the need for cognitive tests or imaging methods (Wolters et al., 2019).

In addition to using each hormone as biomarker individually for assessing severity of cognitive impairment, the value of combined detection of FT3, FT4, and TSH in distinguishing PD patients with and without cognitive impairment is another notable finding of this study. Our study is preceded by other endeavors utilizing TSH, FT3, and FT4 as predictors of psychological and cognitive disorders such as bipolar II depression (Lai et al., 2021). By incorporating multiple thyroid hormone parameters, we obtained a more robust evaluation of the link between thyroid hormones and cognitive function, as revealed by the high sensitivity, specificity, and AUC. This approach may have practical implications for the clinical assessment of cognitive impairments in PD patients.

It is crucial to acknowledge the limitations inherent in this study. Firstly, the adoption of a cross-sectional design restricts our ability to establish a causal relationship between thyroid hormone levels and cognitive impairments. To gain deeper insights into the temporal dynamics of these associations, future longitudinal studies are imperative. Furthermore, the relatively modest sample size employed in our study may curtail the generalizability of the findings. Conducting larger-scale studies is necessary to validate and expand upon our results. In our univariate analysis, gender was not a risk factor for cognitive impairment in PD. Furthermore, in the multivariate analysis presented in Table 2, gender was also not an independent risk factor in our sample. This may be related to our sample size. However, future work will include a larger sample size to verify this. Ultimately, unraveling the underlying mechanisms that link thyroid hormones to cognitive impairments in PD holds the potential to inform the development of targeted therapeutic strategies, thereby enhancing patient outcomes.

## Conclusions

In conclusion, this study provided supporting evidence on the clinical significance of investigating the intricate interplay between thyroid hormones and cognitive impairments in PD patients. The findings underscore the notable correlations between serum thyroid hormone levels and cognitive dysfunction. This emphasizes the potential utility of these hormone levels as promising biomarkers for the detection of cognitive deficits in individuals with PD.

## Data Availability

The data could not be shared openly, as required by our department. The raw data could be obtained upon reasonable request to the corresponding author.

## Synthesis

Reviewing Editor: Karl Herrup, University of Pittsburgh School of Medicine Decisions are customarily a result of the Reviewing Editor and the peer reviewers coming together and discussing their recommendations until a consensus is reached. When revisions are invited, a fact-based synthesis statement explaining their decision and outlining what is needed to prepare a revision will be listed below. The following reviewer(s) agreed to reveal their identity: Tang Ming-Liang.

The revisions and responses have been thoughtful and thorough. The work is now appropriate for publication in eNeuro.
